# Vision-Driven Kinesthetic Illusion in Mirror Visual Feedback

**DOI:** 10.1177/2041669518782994

**Published:** 2018-06-24

**Authors:** Yuki Ishihara, Kenri Kodaka

**Affiliations:** Nagoya City University, Nagoya, Aichi, Japan

**Keywords:** body perception, multisensory/cross-modal processing, mirror visual feedback, proprioception

## Abstract

In the paradigm of mirror visual feedback, it remains unclear how images of the mirrored hand directly affect the sense of motion of the hidden hand (kinesthetic illusion). To examine this question, we created an original mirror visual feedback setup using a horizontal mechanism of motion for the mirror and the hidden hand, each of which could independently be given a specific velocity. It should be noted that this setup can cause the hand viewed in the mirror to move without the involvement of the visible hand. In the experiment, the participants reported the felt direction of the hidden hand’s displacement (left/right) after 4 s dual movements with quasi-randomized velocities. It was found that the subjective direction of motion of the hidden hand was strongly biased toward the direction of the mirror. Further, anatomical congruency was found to affect kinesthetic illusion for cases where the mirror approaches the visible hand.

## Introduction

Mirror visual feedback (MVF) is a well-known method of modulating the body image using the image of a hand viewed in a mirror, using a specific *mirror box* setup, which was first created during the mid-1990s (Ramachandran & Rogers-Ramachandran, 1996; Ramachandran, Rogers-Ramachandran, & Cobb, 1995). In this setup, the participant places his or her arms one in front of and one behind a stand mirror and then moves the hand placed in front of the mirror (the visible hand). In performing this action, the participant feels that the hand viewed in the mirror is the other hand, hidden behind the mirror, resulting in a sense of motion of the hidden hand (the so-called kinesthetic illusion).

MVF has been used clinically as part of mirror box therapy to promote motor rehabilitation in cases of hemiparesis and hemiplegia (e.g., Dohle et al., 2009; Ramachandran & Altschuler, 2009; Rosén & Lundborg, 2005), although the details of how such therapy can affect recovery processes under these conditions remains unclear (Guerraz, 2015). For example, the effectiveness of mirror therapy differs from patient to patient (Ramachandran & Altschuler, 2009), suggesting that the cognitive process that takes place during MVF has a strong sensitivity to individual sensorial preference. Thus, the identification of the sensorial factors that are critical to yielding kinesthetic illusion during MVF would be especially significant.

Recent neuroimaging studies have shown that the recovery of motor function after stroke passes through a transitional stage, where the contralesional hemisphere overworks (e.g., Rehme, Fink, Von Cramon, & Grefkes, 2011). MVF with bilateral hand motion may influence this stage, although the means by which the related activation affects the recovery process remains unclear. Specifically, it is critical to understand how the inhibitory connection in this early stage from the contralesional to ipsilesional M1 may play a role in adaptive or maladaptive reorganization (Xerri, Zennou-Azogui, Sadlaoud, & Sauvajon, 2014). Furthermore, the visual contribution related to the motor recovery has not yet been fully identified in the context of the aforementioned neuroimaging methods, although some case studies have indicated the significant role of the visual input in reorganizing efference copies of motor output (e.g., Sathian, Greenspan, & Wolf, 2000).

The illusory sense of the motion of the hidden hand during MVF for healthy people was examined in a series of experimental studies by Guerraz et al. which have been taking place since 2012; in these studies, the effect of visible arm displacement (obtained by mechanically flexing or extending the elbow along the sagittal plane) on the kinesthetic illusion was examined in various situations. These studies showed that a passive displacement of the visible arm during MVF affects the perceived velocity of the hidden arm (which remains unmoved) significantly more than is the case in the other blind conditions, where a sense of motion of the hidden arm was orally reported in 98% of all trials with the mirror but only in less than 15% trials without the mirror (Guerraz et al., 2012). Guerraz and colleagues also showed that masking the greater part (more than 84%) of the mirrored image significantly decreased the subjective rating of the kinesthetic illusion (Chancel, Brun, Kavounoudias, & Guerraz, 2016). These are vitally important demonstrations, showing the effectiveness of visual feedback on the kinesthetic illusion. However, it is notable that they do not demonstrate that visual feedback can itself trigger the illusion, because the visual movement of the mirrored hand inevitably occurs together with the physical displacement of the visible hand in the MVF setup.

Further, the proprioceptive afferents of the moving hand have been found to modulate the kinesthetic illusion. Another of Guerraz et al.’s (2012) experiments showed that masking the proprioceptive afferents of the visible hand using vibration significantly decreased the kinesthetic illusion. This effect is caused by the principle of interlimb coupling, occurring between the proprioceptive afferents of the two hands. This coupling effect enables participants to draw circles of the same size in a bimanual drawing task, even with their eyes closed (Metral et al., 2014). Interestingly, in this experiment, the resemblance in the magnitude of the circles during the bimanual drawing task was not greater with visual feedback on mirror than with the blind condition. This interlimb coupling has also been observed in the involuntary arm movements of the hidden hand after long-lasting muscle contraction (the so-called floating arm), where the velocity of the involuntary arm movement tends to match the velocity of the passive displacement of the visible arm in the MVF situation. This effect can be reduced by masking the proprioceptive afferents of the visible arm even where visual feedback is active on the mirror, while masking visual feedback does not reduce this effect so long as the proprioceptive afferents of the visible arm is active (Brun & Guerraz, 2015). These results suggest that the effects of visual feedback do not necessarily play a leading role in the kinesthetic illusion in MVF, contradicting what could be expected. Thus, the concept that the kinesthetic illusion results from a combination of visuo-proprioceptive signals from the two arms and is not purely visual in origin is now widely accepted, as that group has emphasized in their latest study (Chancel, Kavounoudias, & Guerraz, 2017).

It is noteworthy that there has been no convincing demonstration showing that MVF can trigger kinesthetic modulation without the physical movement of the visible hand/arm, although there has been a long history of MVF study. This article is intended to pick out the purely visual contribution to the kinesthetic illusion in MVF, excluding interlimb coupling from the traditional MVF setup. In this goal, only the hand viewed in the mirror was to be manipulated. Thus, we produced an original MVF setup with a horizontal mechanism of motion for two kinds of elements: one being the mirror and the other being the hidden hand. First, horizontal movement of the mirror was introduced to create the movement of the hand image without the involvement of the visible hand. This is simply due to its physical nature; the object reflected in the mirror appears to be placed behind the mirror, at intervals corresponding to the distance between the mirror and the object. Thus, the approach or separation of the mirror to or from the visible hand caused the hand viewed in the mirror to appear to move, even where the visible hand remained static. In particular, the hand viewed in the mirror was perceived to move twice as fast as the mirror itself (it is easy to understand this principle by imagining that the mirror is attracted to the visible hand at a uniform velocity until the mirror exactly aligns with the visible hand: the hand on the mirror travels twice the distance that the mirror itself travels). Second, a passive horizontal displacement of the hidden hand was introduced to examine how the proprioceptive afferents of the hidden hand affects vision-driven kinesthetic illusion.

## Method

In all, 16 undergraduate students (7 males and 9 females; 2 females were left-handed) participated in the experiment. The first eight students participated in exchange for one psychology course credit, while the other eight students received a book of tokens as compensation (2,000 yen). All participants were tested with the following identical device. All experiments were conducted in accordance with the Declaration of Helsinki. The study protocols were approved by the ethics committee of the Nagoya City University.

**Figure 1.**
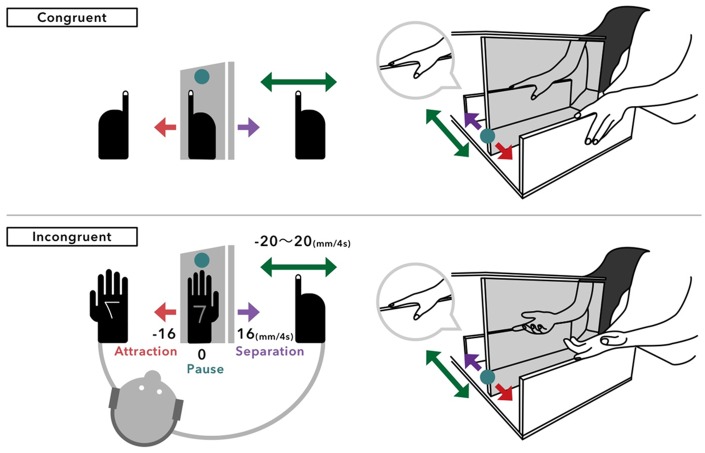
The experimental setup has a horizontal moving mechanism for the mirror and a stand for the right hidden hand. For each trial, the mirror remains unmoved (pause) or moves horizontally 16 mm (for 4 s) toward the right (separation) or left (attraction), while the right stand remains unmoved or moves 4, 8, 12, 16, or 20 mm (for 4 s) toward the right or left. These movements were observed with both hands gripping the stand (congruent) or with the visible hand turned up (incongruent). In this drawing, hand in a bubble depicts the hidden right hand and the image on the grayed plane shows the mirrored image of the visible left hand.

The experimental setup contained one mirror (30 × 45 cm) and two stands for each hand. These stands were initially placed at to the left and right of the mirror, at a distance of 15 cm. The participants were seated in front of the setup such that they could look at the mirror from the left side, with their left and right hands located on the left and right stands. The right side of the mirror was covered with a black cloth to reliably make the right hand invisible. The participant was asked to look at the hand viewed in the mirror at all times and imagine that the hand image was his or her real right hand, with the right hand gripping the right stand and the left hand gripping the stand (congruent) or turned up on the stand (incongruent), as shown in Figure 1.

All participants participated in four sessions; each session was conducted with fixed anatomical congruency (congruent or incongruent). Half of the participants took the congruent session first and third and the incongruent session second and fourth, and vice versa for the remaining participants. In each trial in a specific session, the mirror was paused (pause) or moved horizontally 16 mm to the left, approaching the participant’s visible hand and body-midline (attraction) or to the right, away from them (separation), at a uniform speed for 4 s. In synchrony with this displacement of the mirror, the right stand is paused or moves to the left or right for a distance of 4, 8, 12, 16, or 20 mm (Figure 1). Such a synchronous processing was realized with Arduino Uno (microcontroller board) connecting two linear actuators for both stands. Thus, there were 33 pairs of mirror directions (3) and right-hand movements (11). White noise was temporarily played to mask the sound of the operation of the actuator during the dual motions of 4 s. The noise was played through the wireless headphones worn by the participants. As soon as the noise ceased, the participants were asked to orally report the felt direction (left or right) of the right-hand motion. The trials continued without returning the mirror and the right stand to the original position. A sequence of pairs of mirror directions and right-hand movements was determined pseudorandomly, with the constraint that the mirror and right stand did not go beyond 32 mm and 20 mm from their original positions, respectively. A specific pair of motions (Mirror Direction × Right-Hand Movement) was reproduced 3 times in one session; thus, a total of 99 trials were performed in one session. After the specific session was completed, the mirror and the right stand were returned to their original position, and the posture of the left hand was switched to that corresponding to the other condition of the anatomical congruency.

The collected 66 data for specific mirror direction and anatomical congruency was fitted to a logistic function with a general linear model, the mean shift of which is defined as the point of subjective stationarity (PSS). Thus, PSS indicates at what velocity the hidden hand is felt to be stationary in vision-driven kinesthetic illusion, with specific velocity of the mirror. In addition, PSS indirectly shows a sensorial balance between proprioceptive afferents and visual feedback in creating the sense of motion of the hand. One participant (female) was excluded from the following experimental analysis because she was so influenced by the mirror’s direction that PSS could not be identified.

## Results

**Figure 2.**
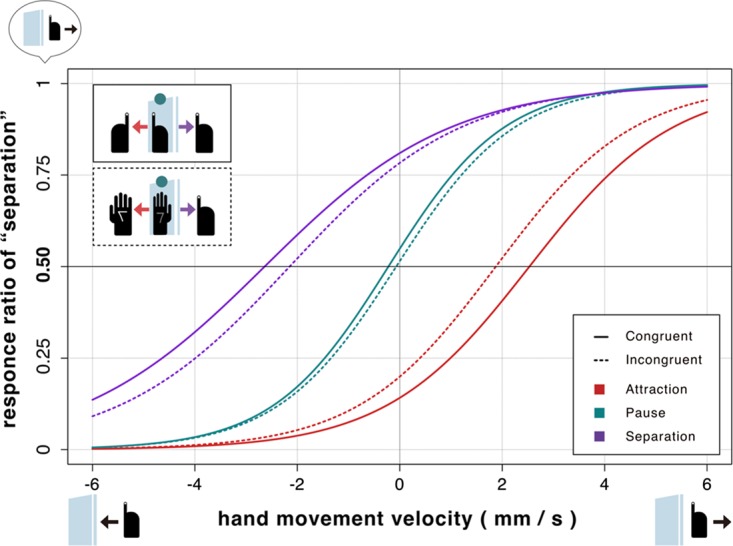
This figure plots psychometric functions (a logistic distribution curve) showing the relationship between the velocities of the right hidden hand (horizontal axis) and the response ratio of participant reports of the hidden hand moving right (vertical axis), for each of three conditions (mirror direction, and attraction/separation/pause). They are depicted separately for the two anatomical conditions (solid/dashed line is corresponding to the congruent/incongruent situation).

Figure 2 plots the relationship between the velocity of the right hidden hand and the response ratio of participant reports of hidden hand moves to the right for each of three mirror conditions (mirror direction, attraction/separation/pause). They are depicted separately for the two anatomical conditions (anatomical congruency, congruent/incongruent). For each graph, the positive or negative direction of the horizontal axis corresponds to the rightward or leftward movement of the hidden right hand (note that the velocity of 8 mm/s in the horizontal axis is equal to the velocity of the hand viewed in the mirror in attraction or separation). Each response ratio is fitted to the psychometric function using a logistic distribution curve, the mean shift of which is defined as a PSS. This graph shows that there is a strong contrast made among the three kinds of PSS.

**Figure 3.**
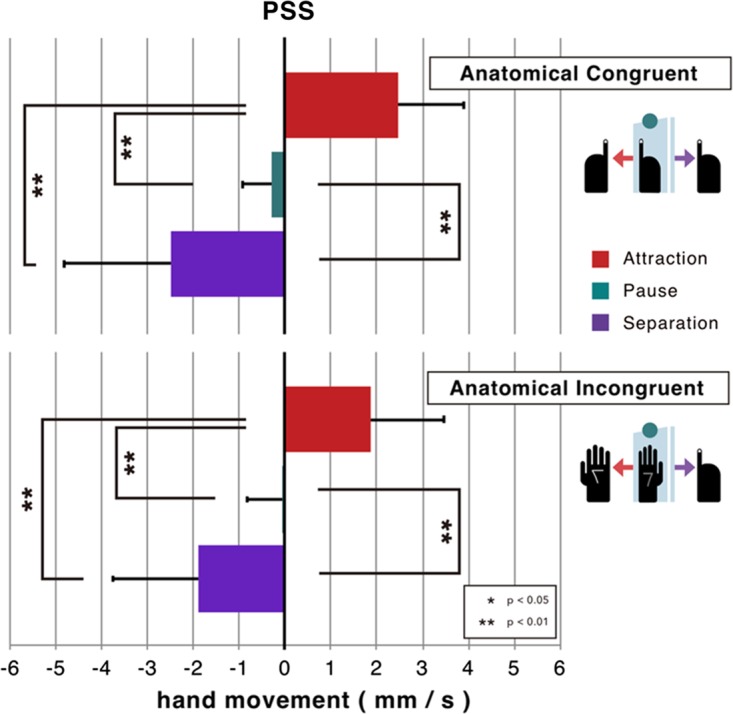
The effects of the movement of the mirror on kinesthetic illusion are compared using the PSS, which was taken as the 50% point of the psychometric function, in each anatomical congruency condition. Statistical significance was calculated using the Holm multiple comparisons method. PSS = point of subjective stationarity.

**Figure 4.**
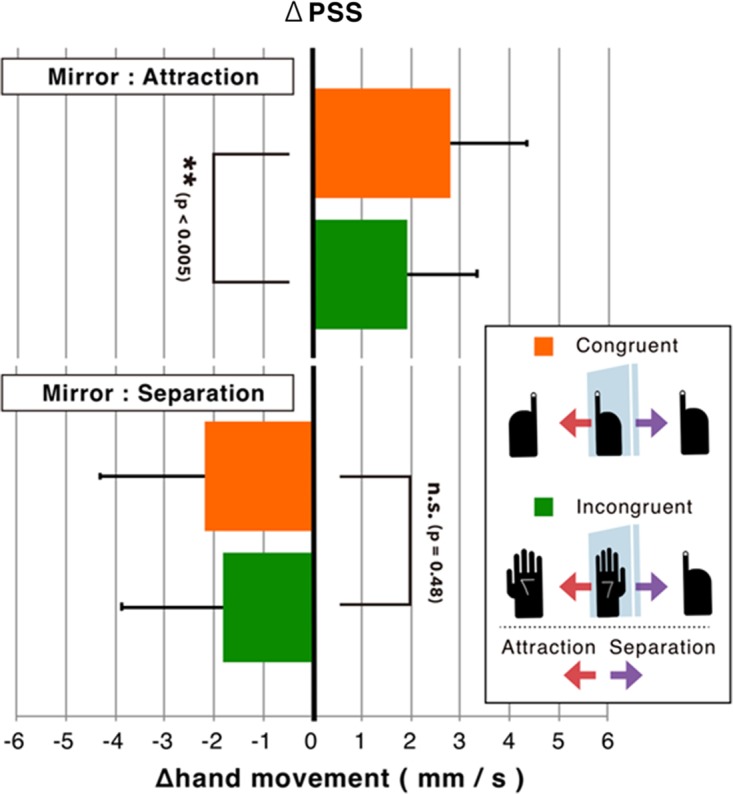
The effects of anatomical congruency on kinesthetic illusion are compared using change in point of subjective stationarity (ΔPSS) for the specific displacement direction of the mirror, calculated by subtracting the PSS in pause from the PSS in attraction or separation for each participant. Statistical significance was only found for attraction (where the mirror approaches the visible hand), using a paired within-subjects *t* test.

A two-way analysis of variance (Mirror Direction × Anatomical Congruency) with within-subject factors revealed a significant main effect for mirror direction, *F*(2, 28) = 25.6, *p* < .001, η^2 ^= .562, but not for anatomical congruency, *F*(1, 14) = 0.43, *p* = .52, η^2 ^= .00. The analysis also found significant interaction between both factors, *F*(2, 28) = 3.35, *p* < .049, η^2 ^= .011; the significant simple main effect of mirror direction was found in both congruent/incongruent situation (*p* < .001). In addition, multiple comparisons using Holm’s (1979) procedure revealed significant differences between all possible pairs from mirror direction, regardless of anatomical congruency (*p*<.01 for all pairs, see Figure 3). These results show the mirrored hand-image movement modulates the illusory direction of the hidden hand movement. On the other hand, post hoc *t* tests comparing two congruency conditions for three mirror movements did not show a significant effect of the anatomical congruency, *t*(14) = 2.09, *p* = .055 for the attractive motion, *t*(14) = 2.09, *p* = .055, *d_D_* = 0.54 for the attractive motion, *t*(14) = −1.57, *p* = .14, *d_D_* = −0.40 for the pause motion, *t*(14) = −1.45, *p* = .17, *d_D_* = −0.38 for the separative motion.

The absolute value of the averaged PSS across participants was around 2.5 mm/s where the mirror moved toward or away from the visible hand at 4.0 mm/s when the hands were anatomically congruent. Because the movement of the mirror doubled the velocity of the mirrored hand, the 8.0 mm/s movement of the hand viewed in the mirror was canceled by the 2.5 mm/s physical movement of the hand to the opposite direction. This means, naively speaking, that the influence of the vision-driven kinesthetic illusion reaches around 30% (2.5/8.0) of the sense of motion that is proprioception driven. This ratio slightly drops to less than 25% in the incongruent situation.

Next, PSS in the moving mirror condition (attraction/separation) was transformed to ΔPSS by subtracting PSS in the static mirror condition (pause) from PSS (in attraction/separation) for each individual. This transformation was designated to compensate for individual differences in the determination of the direction of the subjective hand motion. Note that ΔPSS is defined only for the moving condition (attraction or separation). A two-way analysis of variance (Mirror Direction × Anatomical Congruency) revealed a significant main effect for mirror direction, *F*(1, 14) = 26.4, *p* < .001, η^2 ^= .582, but not for anatomical congruency, *F*(1, 14) = 1.03, *p* = .33, η^2 ^= .002. There was no significant interaction between both factors, *F*(1, 14) = 3.82, *p* = .071, η^2 ^= .011. For further analysis, a paired within-subjects *t* test compared ΔPSS in attraction/separation between congruent and incongruent (anatomical congruency), revealing that the congruent posture shifts PSS in a positive direction stronger than the incongruent posture for attraction, *t*(14) = 3.40, *p* < .005, *d_D_* = 0.878, while there were no significant differences in shifting PSS between congruent and incongruent in separation, *t*(14) = −0.73, *p* = .48, *d_D_* = −0.189, see Figure 4.^1^

## Discussion

The experimental results show that the movement of the mirror modulates PSS, regardless of the direction of movement, where the shifting direction of the PSS corresponds to the displacement direction of the hand viewed in the mirror. This not only means that the movement of the mirrored hand yielded an illusory sense of motion (kinesthetic illusion), even without the physical movement of both hands, but also that the illusory direction of the hidden hand movement was sometimes felt in the opposite direction of the physical movement. Related studies have shown that interlimb coupling in MVF is only effective when the visible hand moves in the same direction as the hidden hand, but not when the visible hand remains unmoved (Brun & Guerraz, 2015; Brun et al., 2015). Thus, the kinesthetic illusion in this experimental setup was purely driven by the visual hand image, excluding the effects of interlimb coupling. Interestingly, another group has shown that seeing the motion of one’s own hand in the video can yield a kinesthetic illusion, even when the physical hand is not actually moving (Kaneko et al., 2015). Our study is the first report showing that this vision-driven kinesthetic illusion can occur, even in MVF.

### Relative Weighting Between Vision and Proprioception

In this experiment, PSS corresponds to the velocity of the hidden hand, which can cancel a vision-driven sense of motion in the hidden hand. In this sense, PSS indirectly represents a sensorial weighting of proprioception and vision information, creating a sense of motion. The relative weighting seen in this result is seemingly consistent with the influence of vision seen in the reaching task paradigm in MVF. In the asymmetric MVF setup, where the distance of the hidden hand from the mirror does not accord with that of the visible hand from the mirror, the proprioception of the hidden hand generally drifts toward the hand viewed in the mirror. This disproportion still remains even after the task of aligning the hidden hand with the hand viewed in the mirror. The early work of Spence and colleagues (Holmes, Crozier, & Spence, 2004) showed that an initial 14 cm (7 cm) discrepancy between the mirrored hand and the physical hand was shortened to 6 cm (3 cm) after the reaching task. That is, the proportion of the influence of vision (to that of the proprioceptive afferents) in determining sense of location in the hidden hand is around 3/7 (around 40%). This ratio decreases when the visuomotor correlation beforehand in making the mirror illusion is insufficient (Holmes & Spence, 2005) or the reaching is done along the radial direction (Snijders, Holmes, & Spence, 2007), while it nevertheless remains around 20% to 40%. Thus, it is interesting that our results have reached a similar conclusion as those of the reaching task paradigm, regardless of the difference in the task; the hand viewed in the mirror surely affects the determining sense of location of that hand, but it is not stronger than the influence of proprioception; the integrated sense of location of the hand is more attracted by the physical position than the visually captured position, in a situation of visual–proprioceptive conflict.

### Relationship With Body Ownership Illusion

Our results show that anatomical incongruent situation also yields kinesthetic illusion, regardless of mirror direction. ΔPSS was introduced to compare the effect of the kinesthetic illusion in terms of anatomical congruency (congruent vs. incongruent). Anatomical congruence significantly magnified the effect of kinesthetic illusion when the mirror moved in a direction approaching the visible hand (attraction), but not in a direction separating from the visible hand (separation).

The positive effects of anatomical congruence on the illusion are consistent with a recent study showing that spatial incongruence between the forearms prior to the illusion induction significantly reduces the sense of motion in the hidden hand (Metral et al., 2015). This spatial or anatomical congruence is an essential requirement for yielding body ownership illusions like the rubber-hand illusion (Botvinick & Cohen, 1998; Kalckert & Ehrsson, 2012). Thus, whether body ownership is appropriately projected on the hidden hand affects kinesthetic illusion, in this setup. On the other hand, the effects of anatomical congruency on ΔPSS are not clear in cases where the mirrored hand is separated from the visible hand (separation). In the paradigm of the rubber-hand illusion, the spatial range where the visuotactile-driven illusion is effective is thought to correspond to the range of the peripersonal space (e.g., Lloyd, 2007). Recent studies have claimed that peripersonal space is made from three separate representations anchored to hand, face, and trunk, which are not fully independent from each other: The peripersonal space around the hand becomes small when the hand separates from its trunk (Serino et al., 2016). Here, it is natural to assume that the body ownership illusion is more active when the mirrored hand image approaches the trunk rather than when it is separated from the trunk. Considering that the mirror in this setup, in attraction (separation), is in a direction approaching to (separating from) the body-midline of the participant, our results suggest that the kinesthetic illusion shares common properties with the body ownership illusion.

### Direction Dependency

The conventional setup for the kinesthetic illusion is what mechanically flexes or extends the elbow along the sagittal plane (e.g., Chancel et al., 2016; Guerraz et al., 2012; Metral et al., 2015). On the other hand, we adopted an azimuthal (left–right) movement of the hand in examining the kinesthetic illusion. We did this simply because only this setup can yield the illusory hand motion without the motion of the visible hand; moving the mirror on the sagittal plane does not create any motion of the hand in the mirror at all.

A previous study showed that the influence of proprioception is relatively weaker in the azimuthal direction than in the radial direction (near–far; Snijders et al., 2007). Our experimental setup may possess a relative advantage in yielding vision-driven kinesthetic illusion, unlike the conventional setup. Examination of whether this vision-driven kinesthetic illusion is applicable to the other directional plane must be left to the future.

## Note

1 We computed the Bayes factor (B) of the ΔPSS comparisons using BayesFactor package for R implemented by Richard D. Morey (http://bayesfactorpcl.r-forge.r-project.org). B for attractive motion was 11.18 (>3) and B for separative motion was 0.331 (<1/3), showing a strong evidence of the congruency effect in the attractive situation and the absence of the congruency effect in the separative situation.
